# Autocrine motility factor promotes endometrial cancer progression by targeting GPER-1

**DOI:** 10.1186/s12964-019-0336-4

**Published:** 2019-03-05

**Authors:** Yiran Li, Yuanhui Jia, Yiding Bian, Huan Tong, Junjie Qu, Kai Wang, Xiao-Ping Wan

**Affiliations:** 1 Department of Gynecology, Shanghai First Maternity and Infant Hospital, Tongji University School of Medicine, Shanghai, China; 2 Clinical and Translational Research Center, Shanghai First Maternity and Infant Hospital, Tongji University School of Medicine, Shanghai, 200040 China

**Keywords:** Endometrial Cancer, Autocrine motility factor (AMF), G protein-coupled estrogen receptor 1 (GPER-1), PI3K signaling pathway, Proliferation, Apoptosis

## Abstract

**Background:**

Autocrine motility factor (AMF) is a critical factor regulating aggressiveness of endometrial cancer (EC). Multiple pieces of evidence indicate that it is through G protein coupled estrogen receptor (GPER) signaling pathway that some growth factors promoted the migration and proliferation of tumor cells. The aim of this study is to explore the role of GPER-1 in AMF mediated regulatory mechanisms of EC recurrence and progression.

**Methods:**

Real-Time Cell Analysis (RTCA) assays were performed to assess whether AMF depends on Autocrine motility factor recepter (AMFR) signaling in EC cells. A genome-wide expression microarray and Yeast Two-Hybrid assay were used to detect AMF and GPER-1 interaction in the context of AMFR depletion, and co-immunoprecipitation and immunofluorescence experiments were performed to confirm the physical interaction. Isobaric Tags for Relative and Absolute Quantification (iTRAQ) analysis was used for the identification of the target pathway activated by AMF-GPER-1 interaction. Cohorts of mice harboring xenografts derived from modified SPEC2 cell lines were treated with or without exogenous AMF to validate the results of previous experiments. Immunohistochemistry was performed to assess AMF and GPER-1 expression in endometrial cancer specimens and normal endometrium.

**Results:**

Our data showed that GPER-1 binds to AMF and the formed complex translocates from the plasma membrane to the cytoplasm. Mechanistic investigations demonstrated that interaction between AMF and GPER-1 triggers phosphoinositide-3-kinase signaling and promotes EC cell growth. More importantly, through animal experiments and human tissue experiments, we found that AMF contributes to GPER-1-mediated EC progression, which is consistent with the above observations.

**Conclusions:**

Our work not only delineated the regulatory mechanisms of endometrial cancer progression by AMF-GPER-1-AKT signaling cascade but also laid the foundation of targeting this pathway for treating endometrial cancer.

**Electronic supplementary material:**

The online version of this article (10.1186/s12964-019-0336-4) contains supplementary material, which is available to authorized users.

## Plain English summary

Traditional surgery plus radiotherapy or chemotherapy and existing targeted therapies have failed to significantly improve the survival rate of recurrent endometrial cancer (EC). Thus, identifying the recurrence and progression mechanisms that modulate EC is clinically important. Although AMFR (autocrine motility factor receptor) is well known as a conventional receptor of AMF, which is a protein secreted by tumor cell stimulating tumor motility. Herein, we showed that autocrine motility factor (AMF) binds G protein-coupled estrogen receptor 1 (GPER-1) and then translocates from the plasma membrane to the cytoplasm. This binding complex directly triggers phosphoinositide-3-kinase signaling and promotes EC cell growth in vitro. Furthermore, we observed that AMF accelerated GPER-1-mediated EC progression in a mouse model. Meanwhile we also observed the significant correlation between AMF and GPER-1 in human EC specimens. On the basis of these data, the AMF-GPER-1 interaction might be a novel key molecular target for the therapeutic management of EC patients who experience progression and recurrence.

## Background

Endometrial cancer (EC) is the most common malignancy of the female reproductive system, with a 3% risk of occurrence. In addition, relatively uncommon, 5 to 10% of EC patients were only 45 years old or younger. Thus, EC is a major threat to a woman’s life and reproductive health [1]. Although most patients with EC are diagnosed early, the median survival time is only 8–12 months, indicating the poor efficacy of treatment in improving survival [2]. In recent years, targeted therapy has attracted increasing attention due to its high specificity, absence of surgical injury and ability to avoid the general toxicity of radiotherapy and chemotherapy [3]. Nonetheless, targeted therapy for the treatment of EC has failed to achieve satisfactory efficacy with an objective response rate < 10%, suggesting that the molecular mechanisms that promote EC progression are still unclear [4].

G protein-coupled estrogen receptor 1 (GPER-1), a seven transmembrane receptor, could mediate tumor cell proliferation via its rapid non-genomic estrogenic responses in female cancer [5]. Other than exacerbated proliferation, GPER-1 is involved in several hallmarks of cancer, including stimulated migration and invasion, the metabolic reprogramming, and induction of angiogenesis, in prostate cancer, lung cancer, and thyroid carcinoma [6]. Multiple studies have demonstrated that GPER-1 mRNA and protein levels are significantly upregulated in breast, endometrial, ovarian and thyroid cancers [7]. Albanito and Pandey have proven that EGF, CTGF and other growth factors that are secreted by breast and ovarian cancer cells activate GPER signaling pathway, which promotes the migration and proliferation of tumor cells [8, 9]. Moreover, the G protein signaling inhibitor PTX and the PI3K signaling inhibitor wortmannin suppress EC cell proliferation [10]. Although studies have demonstrated that the rapid non-genomic effects of GPER-1 promote EC progression [10], interaction of GPER-1 with proliferative factors in the tumor microenvironment has not been clarified. This clarification is crucial for understanding the relationship between metabolic abnormalities and EC as well as the mechanism of anti-estrogen therapy for the treatment of EC.

Autocrine motility factor (AMF) is produced and secreted by tumor cells and activates the downstream PI3K, MAPK and JNK signaling pathways [11], which in turn modulate the directional malignancy of solid tumor cells [12–14]. AMF is also known as phosphoglucose isomerase (PGI), which catalyzes interconversion of glucose-6-phosphate and fructose-6-phosphate in glycometabolism [15]. The dual effects of AMF on tumor cells and glucose metabolism coincide with the dual properties of endometrial tumor proliferation and metabolic abnormalities in EC [3, 16]. Apart from these findings, our previous studies have proved that AMF can regulate EC cell migration, proliferation and invasion by activating the downstream MAPK pathway [17]. However, recent studies have confirmed that the AMF receptor, i.e., AMFR, is not the only receptor that mediates the effect of AMF. For example, AMFR-null Leukemia cells can be induced by AMF stimulation to differentiate [18]. Also, Vitaly et al. demonstrated that AMF binds to HER2 to promote the migration and invasion of breast cancer cells via activated MAPK/ERK signaling pathway [19, 20].

In this study, we show that in human EC cells, AMF binds to the membrane receptor GPER-1, induces the transfer of the AMF-GPER-1 complex to the cytoplasm, activates the downstream PI3K pathway, and promotes tumor cell proliferation and apoptosis. Our data demonstrate that this interaction between AMF and GPER-1 accelerates the progression of this malignancy in the EC microenvironment. These findings provide a reliable experimental basis for molecular targeted therapy, which in turn deepens our understanding of the intrinsic mechanisms underlying the prognosis of EC.

## Methods

### Reagents and antibodies

Purified rabbit PGI was purchased from Sigma for exogenous AMF/PGI stimulation (cat. no. P9544). Recombinant mouse PGI for mouse injection was from Biorbyt (orb 245,854). Mouse monoclonal anti-AMFR (ab76841), rabbit polyclonal anti-AMF (ab86950), rabbit polyclonal anti-GPR30 (also known as anti-GPER-1, ab39742), rabbit monoclonal anti-Ki-67 (ab16667) and anti-β-actin for use in immunohistochemistry and Western blot analyses were obtained from Abcam, Ltd. (Hong Kong, PR China). Mouse monoclonal anti-AMF (ab66340) from Abcam, Ltd. and rabbit polyclonal anti-GPER (AER-050) from Allomone Labs were used for coimmunoprecipitation analyses. Antibodies against p-AKT, AKT, p-ERK1/2 and ERK1/2 were from Cell Signaling Technology (Shanghai, PR China). Wortmannin (PI3K inhibitor) was purchased from Selleck.cn.

### Cell culture and treatment

Two EC cell lines, Ishikawa (the estrogen-dependent EC cell line) and SPEC-2 (the non-estrogen-dependent EC cell line), were purchased from the American Type Culture Collection (Manassas, VA, USA) and maintained according to the provider’s instructions in DMEM/F12 (Gibco, Auckland, NZ) supplemented with 10% FBS (Gibco, Carlsbad, CA, USA). All cells were grown until confluent and incubated in serum-free medium for 24 h before treatment with various experimental agents. Cross-linking with 3,3′-Dithiobis(sulfosuccinimidyl propionate) (DTSSP) was conducted to identify the interaction between AMF and GPER-1. Endometrial cells were washed with PBS and then exposed to DTSSP for 1 h at 4 °C, and the reaction was terminated by the addition of 20 mmol/L Tris–HCl (pH 7.5) for 15 min at room temperature (RT). Cells were extracted with lysis buffer, and insoluble material was removed by centrifugation. Supernatants were then processed for coimmunoprecipitation. Exogenous AMF/PGI was diluted to 10 ng/ml with PBS to stimulate EC cells.

### Transfection and selection

Transfections of Ishikawa and SPEC-2 cells with the GPER-1 knockdown or overexpression plasmid and the AMFR knockdown plasmid were performed as described in our previous report [10, 17]. Selection was maintained by supplementing the cultures with puromycin (2 mg/ml) to obtain stable cell lines, and several puromycin-resistant GPER-1 knockdown/overexpression and AMFR knockdown clones were harvested by ring selection. The levels of GPER-1 and AMFR were confirmed by RT-PCR and immunoblotting [21]. The shRNA sequences are provided in Additional file 1: Table S1.

### RNA extraction and quantitative real-time polymerase chain reaction (qRT-PCR)

TRIzol reagent (Invitrogen, Life Technologies; Shanghai, PR China) was used to isolate total RNA, and a reverse transcriptase kit (TaKaRa, Dalian, PR China) was used for reverse transcription. Gene expression was detected with the SYBR Green master mix (TaKaRa, Dalian, PR China) on an ABI Prism 700 thermal cycler (Applied Biosystems, Foster City, CA, USA). Gene expression was calculated using the 2ˆ(−ΔΔCt) formula and normalized against β-actin. The oligonucleotide primers are provided in Additional file 1: Table S2. All experiments were performed independently three times.

### Western blotting and coimmunoprecipitation

Western blotting and coimmunoprecipitation were conducted according to routine protocols. Protein was extracted from the cells by suspension in RIPA buffer (1 × PBS, 1% Nonidet NP-40, 0.1% SDS) containing a cocktail of protease inhibitors (11,257,200; Roche Diagnostics, Mannheim, Germany). In addition, for detection of GPER-1, protein was extracted using Mem-PER Eukaryotic Membrane Protein Extraction Reagent (89,826; Pierce, Rockford, IL, USA) containing Complete Mini Cocktail and NE-PER Nuclear and Cytoplasmic Extraction Reagents (78,833; Pierce) from EC tissue and normal endometrial tissue. After BCA protein assays (Pierce), Western blot and coimmunoprecipitation with appropriate antibodies were carried out overnight. Statistical data from three repeated experiments were graphed.

### RTCA analysis for cell migration, invasion and proliferation

RTCA analysis for cell migration, invasion and proliferation was performed according to a routine protocol [22]. For migration and invasion assays, 1 × 10^5^ serum-starved Ishikawa and SPEC-2 cells treated with peptides or peptidomimetics were resuspended in 100 μl of serum-free medium and added to the preequilibrated upper chambers of an xCELLigence CIM plate, and the bottom wells of the plate contained complete medium. In parallel, 2 × 10^3^ serum-starved Ishikawa and SPEC-2 cells were resuspended and loaded into the upper chambers of an xCELLigence CIM plate for proliferation analyses. Cell index values were detected every 30 min for 30 h and 100 h throughout the migration/invasion and proliferation assays, respectively. We used RTCA software v1.2 (Roche Applied Science) to calculate the slopes of the curves at various time points.

### Transwell assay

The protocol for the migration and invasion assays with a transwell system was described previously [17]. The number of cells that had migrated or invaded was determined using MetaMorph image analysis software (Molecular Devices, Sunnyvale, CA, USA), and the result was calculated as the mean ± SD (*n* = 3).

### Gene expression analysis

Total RNA was quantified with a NanoDrop ND-2000 spectrophotometer (Thermo Scientific), and RNA integrity was assessed using the Agilent Bioanalyzer 2100 (Agilent Technologies) (GEO: GSE114362). Total RNA was transcribed to double-stranded cDNA, synthesized into cRNA and labeled with cyanine-3-CTP. The labeled cRNA was hybridized onto a microarray. After the samples were washed, the arrays were scanned with the Agilent Scanner G2505C (Agilent Technologies) according to the manufacturer’s recommendations. Feature Extraction software (version 10.7.1.1, Agilent Technologies) was used to analyze array images to obtain raw data. The G protein-coupled receptors (GPCRs) were screened out from differentially expressed genes (fold change≥2) in SPEC-2/shAMFR vs SPEC-2 cells, and these genes also showed the same variation tendency in Ishikawa/shAMF vs Ishikawa cells. We identified 38 differentially expressed GPCR genes, including 24 upregulated and 14 downregulated genes (IUPHAR/BPS Guide to PHARMACOLOGYDA database; http://www.guidetopharmacology.org/), and the heatmaps were plotted using Mev software. (MeV_4_6_0). Finally, gene interaction analysis of 30 upregulated GPCRs was performed in the String database (https://string-db.org/).

### Yeast two-hybrid (Y2H) screen

For bait construction with human GPER-1, cDNA encoding full-length human GPER-1 was cloned into the PPR3-SUC yeast shuttle cloning vector, and cDNA encoding full-length human PGI/AMF was cloned into the PPR3-N yeast vector. These vectors were introduced into the yeast strain NMY51 using a previously described protocol [23, 24], and independent transformants were pooled. After the samples were respread on selection media (SD/−leu-trp-his-ade+3AT) and positive colonies were obtained, the reporter genes AMF and GPER-1 were used to verify the positive colonies.

### Immunofluorescence and confocal microscopic imaging

For the detection of AMF and GPER-1, cells were seeded onto glass coverslips, fixed with 4% paraformaldehyde for 10 min and permeabilized with 4% Triton X-100 in PBS for 10 min. Cells were sequentially incubated with primary antibody diluted in blocking buffer (ab126587, Abcam Biotechnology) overnight at 4 °C, followed by incubation with secondary antibodies conjugated with Alexa Fluor 568 (Molecular Probes-Invitrogen, Carlsbad, CA, USA) for 1 h at RT in the dark. After the cells were washed with PBS, they were stained with 4′,6′-diamidino-2-phenylindole (DAPI) for 5 min to stain cell nuclei. After extensive washing, the cells were mounted on a glass slide with 80% glycerol, and fluorescence images were obtained using a Nikon A1 (Melville, NY, USA) confocal microscope or analyzed with an Olympus fluorescence microscope using a 400x lens.

### Crystal structures of AMF and GPER-1

The structure of Glucose-6-phosphate isomerase (PDB:1JIQ) was obtained from the RCSB PDB (http://www.rcsb.org/pdb/home/home.do). The structure of G protein-coupled estrogen receptor (UniProt:Q99527) was constructed by homology modeling using the structure of bovine rhodopsin as the template (PDB:1JFP). Homology modeling was carried out using the Molecular Operating Environment (MOE). Structural models of AMF/GPI-GPER-1 complexes (AMF/GPI: red, GPER-1: green) were computed with the docking programs ZDOCK through the ZDOCK server (http://zdock.umassmed.edu/).

### Three-dimensional spheroid culture

The AlgiMatrix 3D Culture System (Invitrogen) was used to construct an artificial bioscaffold to facilitate three-dimensional spheroid formation in GPER-1 knockdown cells with or without exogenous AMF stimulation. Cells were seeded into AlgiMatrix six-well plates, grown to form spheroids for 2 weeks and photographed under a microscope. CellProfiler 2.0 software (http://www.cellprofiler.org/) was used for image processing to detect and quantify individual spheroids.

### Flow cytometric analysis

Whole-cell suspensions were stained with 50 μg/ml PI (propidium iodide) after 70% ethanol fixation and analyzed for cell cycle phase distribution with a BD Biosciences flow cytometer. Cell apoptosis was also quantified using an Annexin V-FITC/PI apoptosis kit (BioLegend, 640,914) according to the manufacturer’s instructions. The number of FITC- or PI-positive cells was determined using a flow cytometer (ImageStreamX MarkII, Amnis, USA). The fluorescence levels of FITC and PI were measured in the FL2 channel (488 nm) and FL4 channel (488 nm), respectively. The results were analyzed using IDEAS Application v6.0 software (Amnis, USA).

### iTRAQ sample preparation and QSTAR elite hybrid LC-MS/MS

Total protein was quantified with a BCA protein assay kit, and protein profiles were determined by sodium dodecyl sulfate-polyacrylamide gel electrophoresis (SDS-PAGE) analysis. Then, protein was digested with trypsin. For labeling, each iTRAQ reagent was added to the respective peptide mixture. In addition, the iTRAQ-labeled peptides were fractionated by an Agilent 1100 HPLC chromatographic instrument. The fractions were finally collected into 10 pools for analysis by liquid chromatography with tandem mass spectrometry (LC-MS/MS, Q-Exactive, Thermo Scientific). Proteome DiscovererTM 2.2 was used for peptide and protein identification through UniProt. Differentially expressed proteins were then identified by examining their fold changes. The threshold set for up- and downregulated proteins was a fold change ≥1.2. Gene Ontology (GO) analysis and Kyoto Encyclopedia of Genes and Genomes (KEGG) pathway analysis were applied to determine the roles of the groups of differentially expressed proteins in B vs A and D vs C, and the statistical significance of enrichment (*p* value) was measured through hypergeometric distribution as follows.

$$ \mathrm{P}=1\hbox{-} \sum \limits_{i=0}^{m-1}\frac{\left(\begin{array}{c}M\\ {}i\end{array}\right)\;\left(\begin{array}{c}N-M\\ {}n-i\end{array}\right)}{\left(\begin{array}{c}N\\ {}n\end{array}\right)} $$

The mass spectrometry proteomics data are available via ProteomeXchange [25] with identifier PXD009780. The result shows the differentially expressed proteins with significant correlations with cell proliferation and the cell cycle. In addition, the GO terms and pathways associated with cell proliferation or the cell cycle are shown with histograms and bubble charts (ggplot2 package of R), respectively. A pathway-pathway network was generated using Cytoscape through the interaction relationship in the KEGG database.

### In vivo experiments and analyses

Forty-eight week-old female athymic nude mice (BALB/c) were obtained from Sino-British Sippr/BK Lab Animal Co., Ltd. (Shanghai, China). The mice were randomly divided into four groups, namely, the control group, control group with AMF stimulation, shGPER-1 group, and shGPER-1 group with AMF stimulation, and each group contained 10 mice. All mouse studies were performed in accordance with animal protocol procedures approved by the Department of Laboratory Animal Science at the School of Medicine, Shanghai Tongji University. Excess anesthesia was used to euthanize mice at the end of the experiment.

For this protocol, SPEC-2 cells with stable knockdown of both AMFR and GPER-1 (shGPER-1) and control SPEC-2 cells with only AMFR stably knocked down (mock) were engineered to stably express firefly luciferase. These cells were resuspended in sterile PBS (50 μl) and injected (6 × 10^5^ cells/per mouse) into the tail veins of 8-week-old female BALB/c mice. Mice were divided into the four groups mentioned above depending on the status AMF stimulation (200 ng/per mouse/per week). Metastasis was determined at 1, 14, 28 and 42 days post-injection by bioluminescence imaging on a Xenogen IVIS-200 system (Caliper Life Sciences, Hopkinton, MA, USA). At day 42, we sacrificed the remaining mice that did not die from their tumors. For each mouse, we resected and counted the number of disseminated tumors that were > 1 mm in diameter. The number of systemic tumor metastases were counted, and we measured the volume of the tumor dissemination. Serial histological sections of the ovaries were processed for hematoxylin-eosin (H&E) staining, and histological examinations of AMF, GPER-1, Ki-67 and p-AKT were performed as described above.

### Patients and tissue samples

For this study, tissues from 99 cases of EC, including 52 cases with type I EC and 47 cases with type II EC, and 50 samples of normal endometria were obtained from surgical procedures performed in the Shanghai First Maternity and Infant Hospital between 2014 and 2016. This project was approved by the Human Investigation Ethics Committee of the Shanghai First Maternity and Infant Hospital, and informed consent was obtained from all patients before the study.

### Immunohistochemistry and immunofluorescence

Paraffin-embedded EC and normal endometrial tissue sections (4 μm) were processed for immunohistochemistry. Firstly, specimens were deparaffinized and dehydrated, and then specimens were incubated with a specific antibody. The last step, specimens were detected using Envision reagents (Boster Bioengineering, Wuhan, China) according to the manufacturer’s instructions. To evaluate the expression of AMF and GPER-1, the staining intensity was scored as 0 (negative), 1(weak), 2(medium) or 3 (strong). The extent of staining was scored as 0 (0%), 1(1–25%), 2(26–50%), 3 (51–75%) or 4 (76–100%). The final score, ranging from 0 to 7, was the sum of the intensity score and the extent score. Samples with a final score no more than 3 were defined “negative”. The immunohistochemical results were assessed by two pathologists who were blinded to the tissue source.

For histological analysis, human EC tissue and normal tissue were fixed in 4% paraformaldehyde (Affymetrix, Santa Clara, CA, USA) and embedded in paraffin. The primary antibodies used for immunofluorescence staining of the human tissues were antibodies against AMF (ab66340, Abcam Biotechnology) and GPER-1 (ab39742, Abcam Biotechnology). Alexa 488- and Alexa 594-conjugated antibodies (Life Technologies, Carlsbad, CA, USA) were used. DAPI staining was performed for the detection of nuclei, and confocal microscopic images were obtained for subsequent analysis.

### TCGA data analysis

AMF and GPER-1 mRNA expression which were normalized by fragments per kilobase of transcript per million mapped reads upper quartile (FPKM-UQ) and clinical data of uterine corpus endometrial carcinoma were downloaded from The Cancer Genome Atlas (TCGA). Kaplan-Meier estimate of relapse-free survival was performed based on AMF and GPER-1 expression. Samples were dichotomized by bottom quantile of each genes. *P*-value was calculated using log-rank test.

### Statistical analysis

Continuous variables are shown as the mean ± SD, and all statistical analyses were performed using Statistical Package for Social Sciences software version 17.0 (Chicago, IL, USA). Volumetric data were assessed using an unpaired Student’s t-test, and one-way ANOVA followed by post hoc LSD test or Dunnett’s test was used for multiple comparisons. Concordance and correlation between AMF and GPER-1 were assessed with chi-square tests. Survival curves were assessed using a standard log-rank test and the Kaplan–Meier method. *P* values< 0.05 were considered statistically significant. All experiments were repeated independently at least three times.

## Results

### AMF induces EC cell proliferation in an AMFR-independent manner

**Fig. 1:**
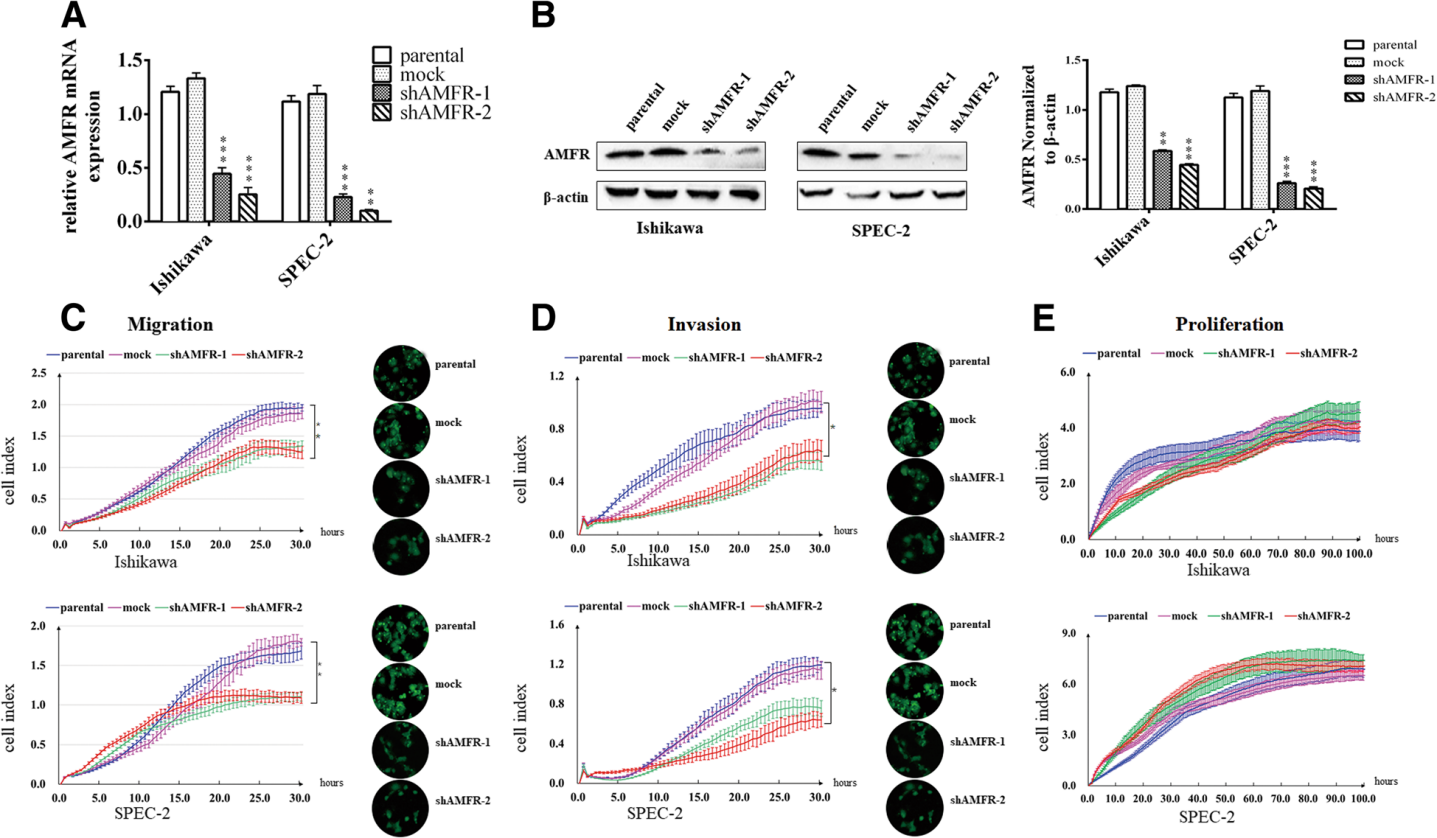
AMF induces proliferation in an AMFR-independent manner in EC cells. **a**. Ishikawa and SPEC-2 cells were stably transfected with plasmid containing AMFR-specific shRNA (shAMFR-1 or shAMFR-2) or control plasmid (mock). Cells were then analyzed by qRT-PCR. **b**. Left, immunoblot analysis for AMFR and β-actin protein expression; Right, quantification of AMFR expression. C and D. RTCA and transwell assays for cell migration (**c**) and invasion (**d**). Cells were seeded onto the upper surfaces of chambers without (**c**) or with Matrigel coating (**d**) and analyzed after 30 h of incubation. Left, Cell index values were quantitated and are expressed as the mean ± SD from three independent experiments; right, Photographs depict the migration or invasion of EC cells (*P < 0.05; **P < 0.01, and ***P < 0.001 compared with the control cells). **e**. Growth curve by RTCA assay. Cells were seeded at a low density (2000 per well) and grown for 100 h (points, mean of triplicate determinations; bars, SD)

It is demonstrated that AMF played a key role in enhancing EC progression. To determine whether AMF depends on AMFR signaling, we initially used the EC cell lines Ishikawa and SPEC-2, which were chosen according to their high AMFR expression levels in a PCR analysis (data not shown), for stable transfection with lentiviral vectors encoding shRNA targeting human AMFR or an empty vector that served as the control. We measured the levels of mRNA and protein expression in the transfectants to examine the efficiency of AMFR silencing, and the results showed that the use of target shRNA sequences against AMFR led to significant depletion of AMFR expression (Fig. 1a and b). Next, migration, invasion and proliferation were measured, and we detected a significant suppression of migration and invasion in the AMFR-silenced cells compared with the empty control cells (Fig. 1c and d). However, knocking down AMFR did not have significant change on cell proliferation (Fig. 1e). These results of depleting AMFR negatively regulating migration and invasion but not proliferation indicate the involvement of another AMF receptor for activation of proliferation.

### AMF interacts with GPER-1 and induces its activation

**Fig. 2:**
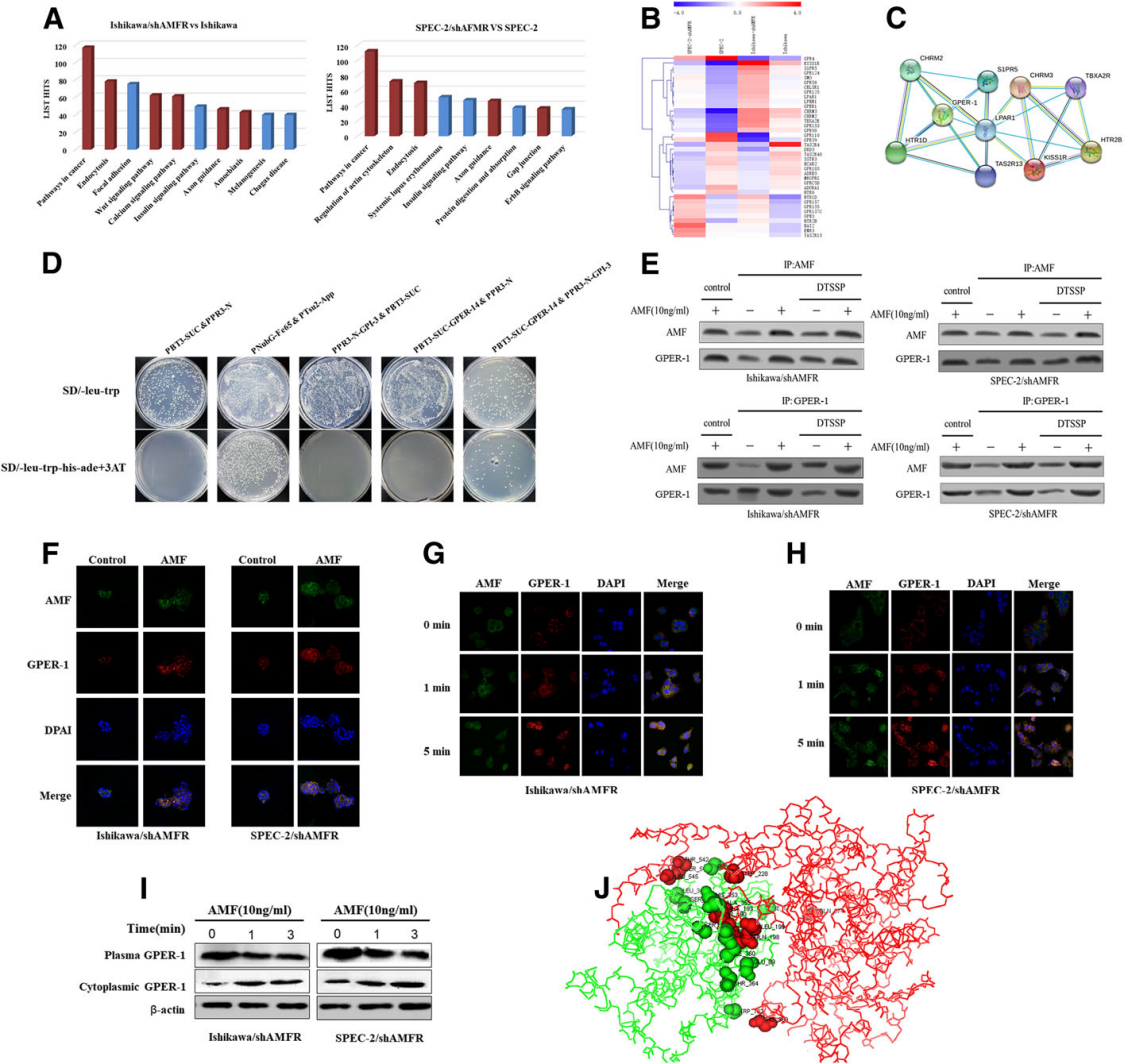
AMF interacts with GPER-1. **a**, A genome-wide expression microarray was used to detect the changes in gene expression by GPCRs under AMFR silencing conditions, and the histogram of the top 10 pathways per the signaling pathway enrichment results is shown. GPCR-related pathways are individually annotated in red. **b** The heatmaps of the same trend changes observed in Ishikawa and SPEC-2 cells (> 1.5-fold) were plotted using Mev software (MeV_4_6_0), including 38 GPCR genes (25 upregulated and 14 downregulated). **c**. Analysis of the gene interactions of 25 upregulated GPCR genes. The results showed that GPER-1 expression was significantly associated with AMF, as shown by the String database. **d**. Cells containing the plasmids encoding PBT3-SUC-GPER-1-14 and PPR3-N-GPI-3 were able to grow in SD medium lacking histidine and adenine. Deletion of the transactivation domain as a negative control completely abolished the growth of clones, and replacement of PNubG-Fe65 and PTsu2-App was used as a positive control in the selective medium. **e**. Serum-starved Ishikawa and SPEC-2 cells were treated simultaneously with AMF and DTSSP for 1 h at 4 °C to cross-link extracellular proteins. Coimmunoprecipitation of lysates was conducted as indicated, and the precipitates were analyzed by Western blot analysis, which allows for the identification of interacting proteins. AMF-treated and cross-linked cells were used for coimmunoprecipitation with anti-AMF (top) or anti-GPER-1 (bottom). **f**. Colocalization of GPER-1 and AMF in serum-starved EC cells, which were treated with exogenous AMF (10 ng/ml) for 24 h. The merge is shown as GPER-1 (red), AMF (green) and DAPI (nuclear stain, blue). Control indicates conditioned medium alone. **g** and **h**. Cells were treated with AMF (1 ng/ml; 0, 1, or 5 min), and the expression of AMF and GPER-1 was detected using immunofluorescence. **i**. Western blots were used to detect the expression of GPER-1 protein in both the cell membrane and cytoplasm to further confirm the results. **j**. Prediction of interaction of AMF-GPER-1 docking, red and green indicate AMF and GPER-1 respectively

Tumorigenic activation by AMF is mediated by its interaction with receptors on the surfaces of target cells [20, 26], such as G-protein family members, including AMFR and HER2 [19, 20]. To determine whether another AMF receptor mediates cell proliferation in EC, we used a genome-wide expression microarray to detect changes in gene expression under AMFR-silencing conditions. Enrichment analysis of the signaling pathways based on gene chip data and the histogram of the top 10 signaling pathways associated with the GPCR are indicated separately in red (Fig. 2a). Combined with previous studies, the results showed that the receptor interacting with AMF may be a GPCR. Next, we identified 38 genes in the GPCR protein family that simultaneously express changes in Ishikawa/shAMFR and SPEC-2/shAMFR cells (Fig. 2b). We hypothesized that expression of another AMF receptor would increase to counteract the loss of AMFR expression and subsequently target upregulated GPCR genes in the analysis. We performed corresponding protein interaction analysis in String to clarify the interactions between these 25 upregulated proteins. According to the chart, GPER-1 is located at the core of the protein interactions, suggesting that GPER-1 may be the most relevant gene to AMF after AMFR knockdown (Fig. 2c). To further verify whether AMF interacts with GPER-1, we first established a Y2H assay to detect the AMF-GPER-1 interaction. The bait plasmid PBT3-SUC-GPER-1-14 and prey plasmid PPR3-N-GPI-3 were transformed into the yeast strain NMY51, and a self-activation test was performed with selective plates (Additional file 1: Figure S1). As expected, only cells containing both plasmids encoding PBT3-SUC-GPER-1-14 and PPR3-N-GPI-3 and not the control cells were able to grow in SD medium lacking histidine and adenine. Deletion of the transactivation domain as a negative control completely abolished the growth of clones, and replacement of PNubG-Fe65 and PTsu2-App was used as a positive control in selective medium, indicating the specificity of the interaction (Fig. 2d).

Because the data indicate that AMF binds to GPER-1, we tested the interaction of exogenously added AMF with GPER-1 using a membrane impermeable cross-linker (DTSSP), which establishes extracellular associations of proteins. After exogenous AMF stimulation, the interaction between AMF and GPER-1 was clearly observed by reciprocal coimmunoprecipitation analysis (Fig. 2e, top and bottom). In addition, immunofluorescence revealed the colocalization of GPER-1 and AMF (Fig. 2f). As GPER-1 activation is induced by estrogen following GPER-1 localization from the cell membrane to the cytoplasm [27], we determined whether AMF induces activation of GPER-1. As shown in Fig. 2g and h, GPER-1 was localized in patches at the cell surface without exogenous AMF stimulation, and surprisingly, the distribution of GPER-1 in the cytoplasm was more readily observed within 5 min of AMF treatment, indicating the specificity of this response and that AMF induces activation of GPER-1 within a very short time frame of 5 min. Furthermore, we evaluated expression of the GPER-1 protein both at the cell membrane and in the cytoplasm to confirm the above results (Fig. 2i). Taking advantage of data obtained from the crystal structures of AMF and GPER-1 by previous studies [28, 29], we predicted physical interactions between AMF and GPER-1 using ZDOCK software (http://zdock.bu.edu) (Fig. 2j and Additional file 1: Table S3).

### AMF activates the PI3K signaling pathway to promote EC cell growth via GPER-1

**Fig. 3:**
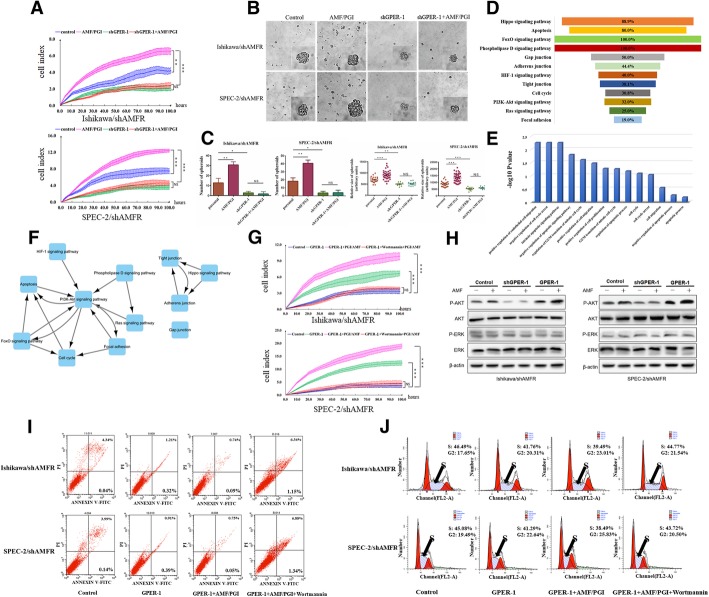
AMF activates the PI3K signaling pathway to promote EC cell growth via GPER-1. **a**. Growth curve by RTCA assay. Cells were seeded at a low density (2000 per well) and grown with exogenous AMF stimulation for 5 days. Fresh medium with AMF (10 ng/ml) was provided every day (points, mean of triplicate determinations; bars, SD). **b**. shGPER-1 and control cells were seeded in 3D culture for spheroid formation and were photographed at day 14 in culture (representative images are shown; 400× for the inserts, 200× for all others). **c**. Quantification of the number (left) and relative size (right) of the spheroids. **d**. For the identification of the target proteins upregulated by GPER-1 overexpression with the simultaneous silencing of AMFR, proteomics analysis using an iTRAQ reagent and QSTAR Elite Hybrid LC-MS/MS was performed. The labeled digests were analyzed using Nano LC-MS/MS. The distribution of enriched KEGG pathways associated with upregulated proteins is shown. **e**. The upregulated expressed proteins were enriched for biological process. **f**. The pathway interaction network was based on KEGG pathway enrichments and is shown in the middle as interactions with high confidence scores. **g**. The effect of wortmannin on AMF-GPER-1-induced cell proliferation. Cells treated as described above were then counted using RTCA assays. Data represent the mean ± SD of the three independent experiments. **h**. EC cells with overexpression of GPER-1 or shGPER-1 and AMFR silencing were serum-starved for 16 h and stimulated with purified AMF (10 ng/ml); p-ERK and p-AKT levels were monitored by Western blot after 15 min, and β-actin was used as a loading control. **i** and **j**. Cell apoptosis and cell cycle profiles were analyzed by fluorescence-activated cell sorting (FACS). (*P < 0.05, **P < 0.01, ***P < 0.001; NS, not significant, one-way ANOVA)

Previous in vitro cell proliferation assays have shown that EC cell proliferation is promoted by AMF [17]. Notably, GPER-1 is expressed and activated in response to estrogen stimuli for EC cell growth [30]. Therefore, we hypothesized that AMF interacts with GPER-1, resulting in accelerated growth of EC. Initially, we established stable cell lines with either GPER-1 overexpression or knockdown in Ishikawa/shAMFR and SPEC-2/shAMFR cells, and Western blot analysis and qRT-PCR confirmed successful overexpression or knockdown of GPER-1 (Additional file 1: Figure S2 and Figure S3). As expected, a cell proliferation assay showed that with endogenous AMF stimulation, silencing of the GPER-1 gene caused stagnation of cell proliferation, whereas control cells continued to proliferate normally. The results motivated us to explore the endogenous interaction of AMF with GPER-1 and investigate the effect of GPER-1 silencing on cell proliferation. AMF knockdown decreased the cell proliferation rate, resulting in a reduction in cell number (Fig. 3a). To further confirm these results, we employed a three-dimensional spheroid culture system to mimic the physiological conditions of tumor growth with AMF stimulation and compared the spheroid-forming ability of the GPER-1 knockdown cells with that of the control cells (Fig. 3b). Consistently, both the size and number of spheroids formed were reduced in cells transfected with shGPER-1 compared with the spheroids in the mock controls (Fig. 3c Left and Right). Taken together, these results suggested that AMF might affect endometrial tumor growth via GPER-1.

To investigate the specific mechanisms involved, we performed proteomics analysis using the iTRAQ reagent and QSTAR Elite Hybrid LC-MS/MS to assess GPER-1 overexpression with simultaneous silencing of AMFR. KEGG pathway analysis and GO analysis were applied to determine the role of the group of differentially expressed proteins in the GPER-1 overexpression condition vs control condition. We analyzed upregulated proteins, and the results of pathway enrichment analysis of the proteins identified 12 pathways associated with cell invasion and proliferation (Fig. 3d). Based on GO terms, upregulated proteins were significantly correlated with cell apoptosis, cell proliferation and cell cycle (Fig. 3e). Through pathway-pathway interaction analysis, we further confirmed that PI3K most likely participates in the AMF-induced signaling linked to GPER-1 (Fig. 3f). To confirm these results, we performed Western blot analyses to assess levels of p-AKT and p-ERK1/2 induction by AMF in shGPER-1-transfected and GPER-1-overexpressing Ishikawa/shAMFR and SPEC-2/shAMFR cells (Fig. 3h). Next, we used the PI3K inhibitor wortmannin to perform cell proliferation, apoptosis and cell cycle analyses. As shown in Fig. 3g and i, the PI3K signaling inhibitor wortmannin overcame the AMF-induced GPER-1 promotion of cell proliferation and apoptosis, as well as cell cycle progression, by arresting cells in the S-to-G2 phase transition compared with control cells without the inhibitor (Fig. 3j). Consistent with the earlier results (Fig. 3a and b), the existence of the AMF/GPER-1/PI3K axis in EC cells, which mediates cancer cell growth to assist EC progression, was confirmed.

### AMF promotes tumorigenicity mediated by GPER-1 in vivo

**Fig. 4:**
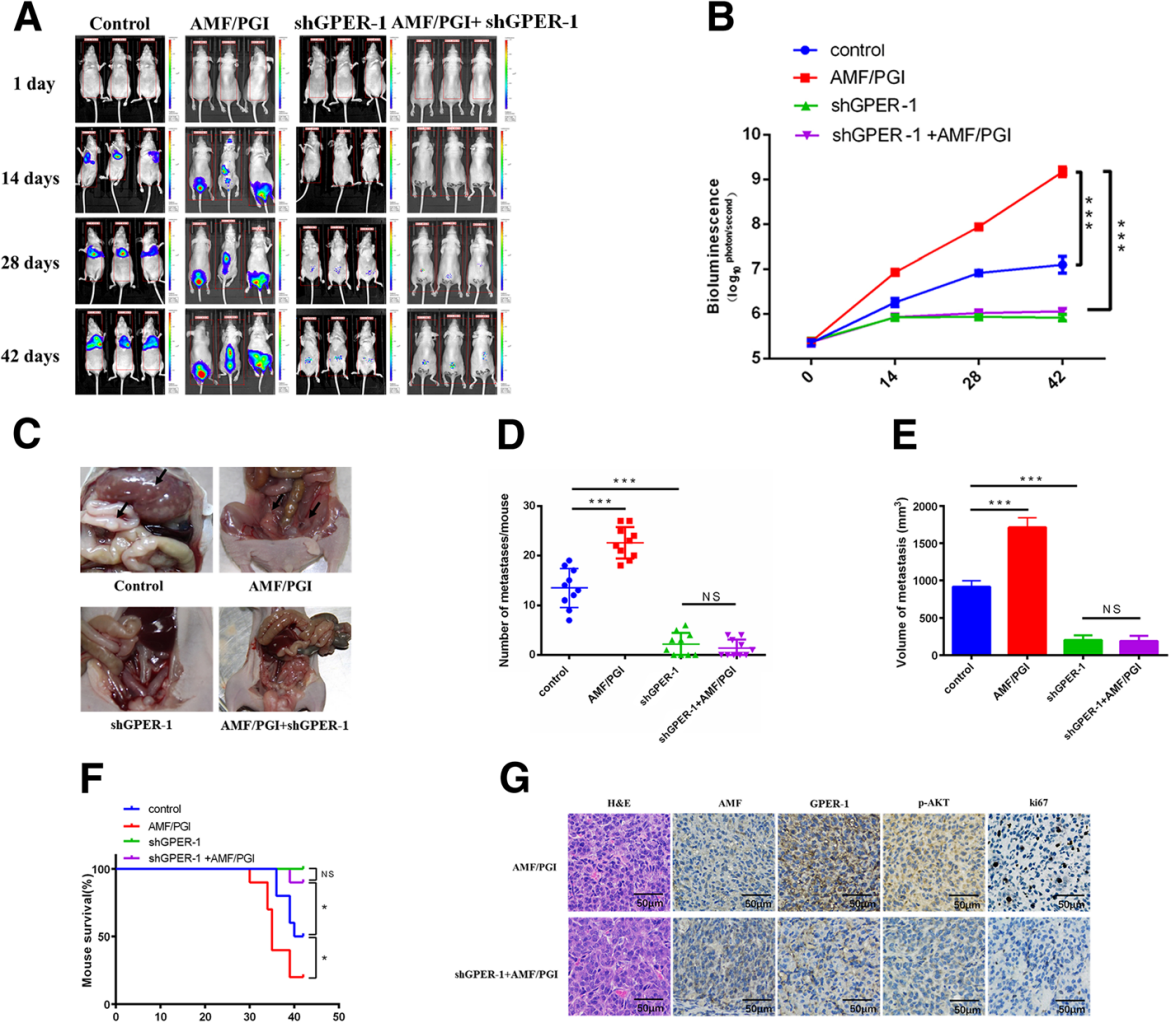
The effects of silencing GPER-1 on AMF-induced tumorigenicity in vivo. Under the indicated conditions, mock or shGPER-1 cells with AMFR deleption, containing luciferase were injected (6 × 10^5^ cells per mouse) with or without exogenous AMF, and 8-week-old nu/nu female mice were randomly allotted to four groups (*n* = 10 mice per group). **a**. Tumor metastasis over a 6-week period by bioluminescence analysis. **b**. Quantitative analysis of metastatic cells based on bioluminescence imaging. The means and 95% confidence intervals (error bars) are presented; ***P < 0.001. *P* values were calculated using a two-sided Student’s t-test. p/sec/cm2/steradian. **c**. Macroscopic view of intraperitoneal injection-derived tumor metastasis. Black arrow, tumor metastasis. **d**. Tumor metastasis per mouse was calculated and measured. **e**. Average volumes of tumor metastasis in the four groups. *P* values were calculated using two-sided Student’s t-tests; ***P < 0.001; NS, not significant. **f**. Kaplan–Meier analysis of mouse survival after xenograft implantation. *P* values were calculated using two-sided log-rank tests (*P < 0.05, NS, not significant). **g**. Representative H&E histopathology analyses of ovarian metastases in mice; AMF, GPER-1, Ki-67 and p-AKT expression was detected by immunohistochemistry (magnification, 200×)

To investigate whether the AMF/GPER-1 axis facilitates EC progression in vivo, we inoculated SPEC-2/shAMFR cells, namely, mock and shGPER-1 cells, into nude mice via the tail vein. The mice were monitored for 42 days with or without tail vein injections, and tumor growth was serially monitored by bioluminescence imaging during this period. We observed an increased tumor formation ratio in mice injected with AMF, and the silencing of GPER-1 overcame AMF-induced tumor progression (Fig. 4a and b). To confirm this observation, the numbers and weights of the metastases were determined by pathological and anatomical analysis (Fig. 4c to e). Our results indicated that stable knockdown of GPER-1 substantially abrogated the tumor growth induced by AMF in EC. As a result, the survival of shGPER-1-treated tumor-bearing mice was prolonged significantly (Fig. 4f). With H&E and immunohistochemical staining, we examined the tumor tissues to further verify that the AMF-induced effect interacted with GPER-1. As shown in Fig. 4g, GPER-1 and phospho-AKT levels, as well as the ki-67 levels, were lower in shGPER-1 tumors than in control tissues. In summary, our experiments suggest that GPER-1 mediates the AMF promotion of EC progression via the PI3K pathway in vivo.

### AMF expression is correlated with GPER-1 expression in clinical samples

**Fig. 5:**
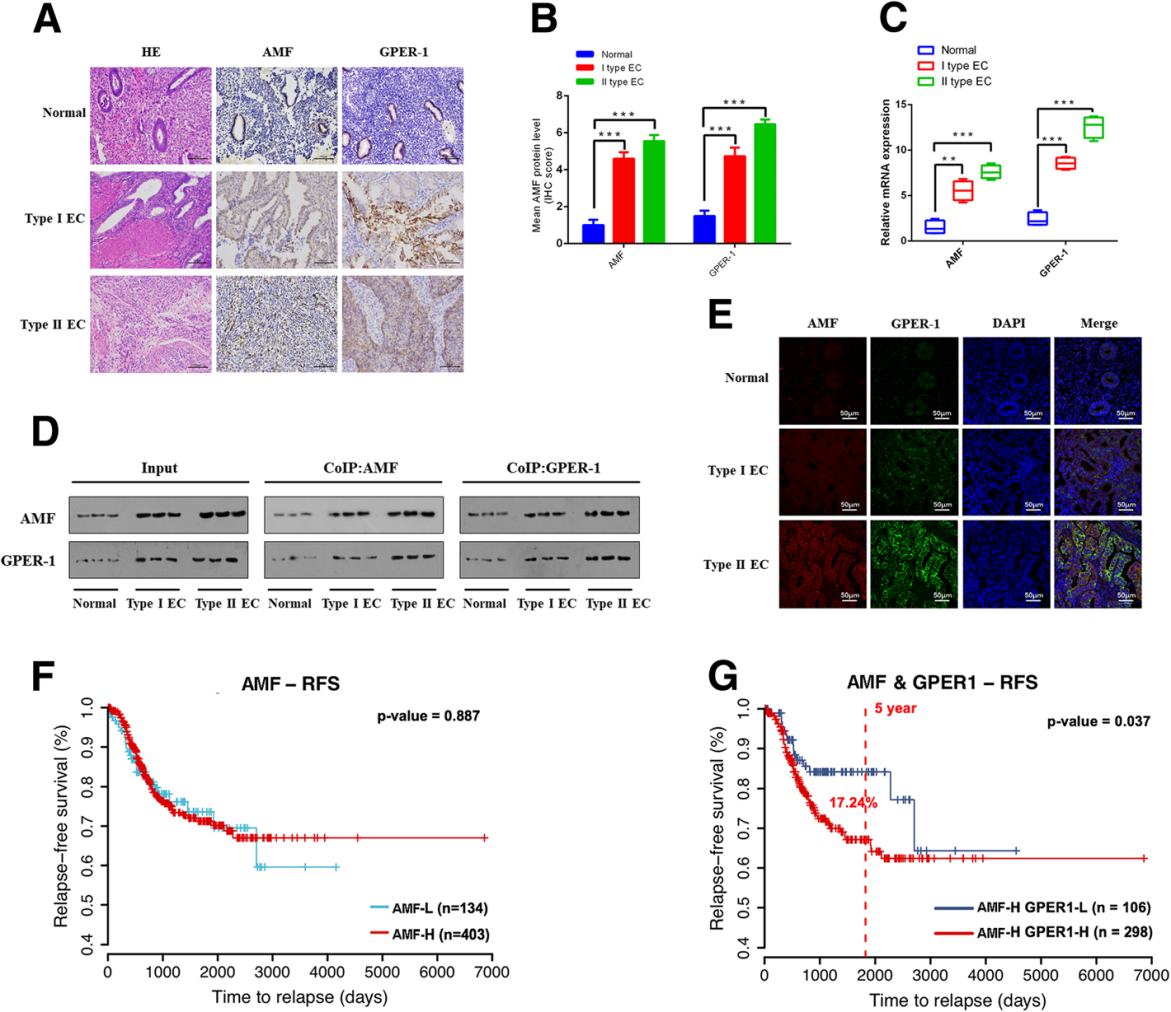
AMF expression correlated with GPER-1 expression in clinical samples. **a**. Immunohistochemical analysis of AMF and GPER-1 expression in the normal endometrium (*n* = 50) and EC, including type I EC (*n* = 52) and type II EC (*n* = 47). **b**. Statistical summary of the immunostaining intensities of AMF and GPER-1 in tissues; AMF and GPER-1 are highly expressed in endometrial cancer (***P < 0.001), and their expression levels are positively correlated (r = 0.92, *P* < 0.01). **c**. AMF and GPER-1 expression in EC tissue specimens and normal endometria was assessed by qRT-PCR and normalized to β-actin expression (**P < 0.01, ***P < 0.001, unpaired Student’s t-test). **d**. The interaction between AMF and GPER-1 was detected by coimmunoprecipitation, and each group included three cases. **e**. Immunofluorescence was used to confirm the AMF-GPER-1 colocalization in clinical specimens. **f**. Kaplan–Meier analysis of TCGA uterine corpus endometrial carcinoma showing the correlation between PGI expression and relapse-free survival. **g**. Kaplan–Meier analysis showing the correlation between GPER-1 expression and relapse-free survival in PGI high expressed samples. (*P*-value calculated by log-rank test)

AMF and GPER-1 expression was evaluated in normal endometrial tissues (50 samples), type I EC tissues (52 samples) and type II EC tissues (47 samples) using immunohistochemistry (Fig. 5a) and qRT-PCR (Fig. 5c). Expression levels of AMF and GPER-1 were markedly higher in EC tissues than in normal endometrial tissues (*P* < 0.001) (Fig. 5b and c). As shown in Fig. 5d, coimmunoprecipitation analyses indicated that GPER-1 expression was positively correlated with AMF expression in EC tissues (Fig. 5d), and immunofluorescence was performed to further confirm the AMF-GPER-1 relationship (Fig. 5e). In conclusion, the data from clinical samples show that AMF might interact with GPER-1, resulting in EC progression, which supports the previous results of both in vitro and in vivo. To further testify our results, we analyzed the correlation between the expression of AMF-GPER-1 and endometrial cancer based on TCGA. We found that patients with high expression level of AMF did not affect the patient’s relapse-free survival (Fig. 5f), while both high expression levels of AMF and GPER-1 have poor prognosis that the 5-year survival rate decreased by 17.24% (*p* = 0.037) (Fig. 5g), which suggested that AMF-GPER-1 closely involved in the progression of endometrial cancer.

## Discussion

AMF/PGI, contributing to the aggressiveness of various types of tumors [19, 31], has been assessed with respect to biochemical characteristics and cytokine activity in previous studies. Our previous work has demonstrated that AMF/PGI performed an oncogenic role in EC [17]. Here for the first time we demonstrated that besides AMFR, GPER-1 is another potential receptor for AMF. AMF may interact with GPER-1 to affect EC growth, including cell proliferation, cell cycle and apoptosis, by activating the PI3K signaling pathway.

It is well known that AMF can directly bind to AMFR to perform important roles in tumor development and progression. However, recent studies suggest that besides AMFR, AMF is also able to bind to HER2 to promote the cancer cell migration and invasion in breast cancer [18]. Similarly, in the current study we identified GPER-1, as another novel interacting partner of AMF (Fig. 2d and Additional file 1: Figure S1), physically associated with AMF to promote EC progression. Importantly, we demonstrated that co-expression of AMF and GPER-1 induced the two protein complex to redistribute and translocate to the cytoplasm within 5 min post exogenous AMF treatment, strongly suggesting that AMF interacts with GPER-1, and facilitates AMF activity in the cytoplasm via the AMF-GPER-1 complex, thereby promoting tumor progression.

Estrogen (E2) and several types of Selective Estrogen Receptor Modulators (SERM) can interact with GPER-1 to activate rapid nongenomic signaling events by triggering downstream PI3K/AKT or MAPK pathway [32]. Indeed a growing body of evidence showed that GPER-1 is strongly associated with different cancer cells proliferation through PI3K signaling pathway, including ovarian cancer and endometrial cancer [9, 33]. Several studies demonstrated AMF can also activate downstream PI3K, MAPK and JNK signaling pathways [11]. Interestingly, our data indicate that silencing of the GPER-1 gene can overcome the EC cell proliferation induced by AMF, suggesting that AMF may cooperate with GPER-1 to ultimately stimulate EC cell growth (Fig. 3a to c). In addition, our results demonstrate that AMF-GPER-1 complex induced cell proliferation, cell cycle and apoptosis may be mediated by activating the PI3K signaling pathway (Fig. 3h, i and j), suggesting that AMF may bind to GPER-1 to promote EC progression also through PI3K signaling pathway. Overall, the function of estrogen and AMF may overlap, which suggests a competitive relationship between them. However, our results show that AMF may affect EC growth through GPER-1 both in the estrogen-dependent endometrial cancer cell line Ishikawa and the non-estrogen-dependent endometrial cancer cell line SPEC-2. Therefore, similar to the redundant functions of ERa and ERβ in PI3K pathway associated with sperm survival [34], and the redundant functions of estrogen-related receptors ERRγ and ERRβ in promoting reprogramming of mouse fibroblasts [35], it is possible that estrogen and AMF do not compete for specific receptors, but instead function redundantly during EC tumorigenesis and progression. The activation of GPER-1 downstream pathways is coupled with EGFR transactivation via Src-mediated metal-loproteinases upregulation, which driving the release of HB-EGF and eventually activating EGFR [6].

On the basis of the in vitro study that a novel interaction between AMF and GPER-1 promoted EC growth, we propose that GPER-1 silencing may inhibit AMF-stimulated EC tumorigenesis in vivo. As expected, exogenous AMF stimulation alone strongly accelerated tumor growth, and this pro-tumor activity was abolished when it was combined with GPER-1 deletion using in vivo tumor burden assay (Fig. 4a to c). Our results indicate that loss of GPER-1 suppresses tumor development and increases survival considerably in the context of AMF-induced EC tumorigenesis and suggest that the AMF-GPER-1 interaction can accelerate tumor progression both in vitro and in vivo (Fig. 4f).

Although studies have proved that in EC patients, high levels of GPER-1 expression predict poor survival [36, 37]. Meanwhile AMF was also identified as an important factor that promotes the occurrence and progression of EC in patients [17]. In the current study we demonstrated that the AMF-GPER-1 pathway facilitated endometrial cancer growth and progression both in vitro and in vivo. Importantly, we observed that the expression level of AMF and GPER-1 are higher in EC tissues than that in normal tissues (Fig. 5a to c). And a significant correlation was observed between the expression level of AMF and GPER-1 in human EC specimens (Fig. 5d and e). TCGA data analysis strongly supported our findings that high AMF-secreting GPER-1-positive cancer cells initially and intrinsically have the advantages of growth and malignancy. Therefore, we propose that AMF might serve as a novel therapeutic target in patients with GPER-1 positive EC, and this target could provide new treatment strategies.

## Conclusion

**Fig. 6:**
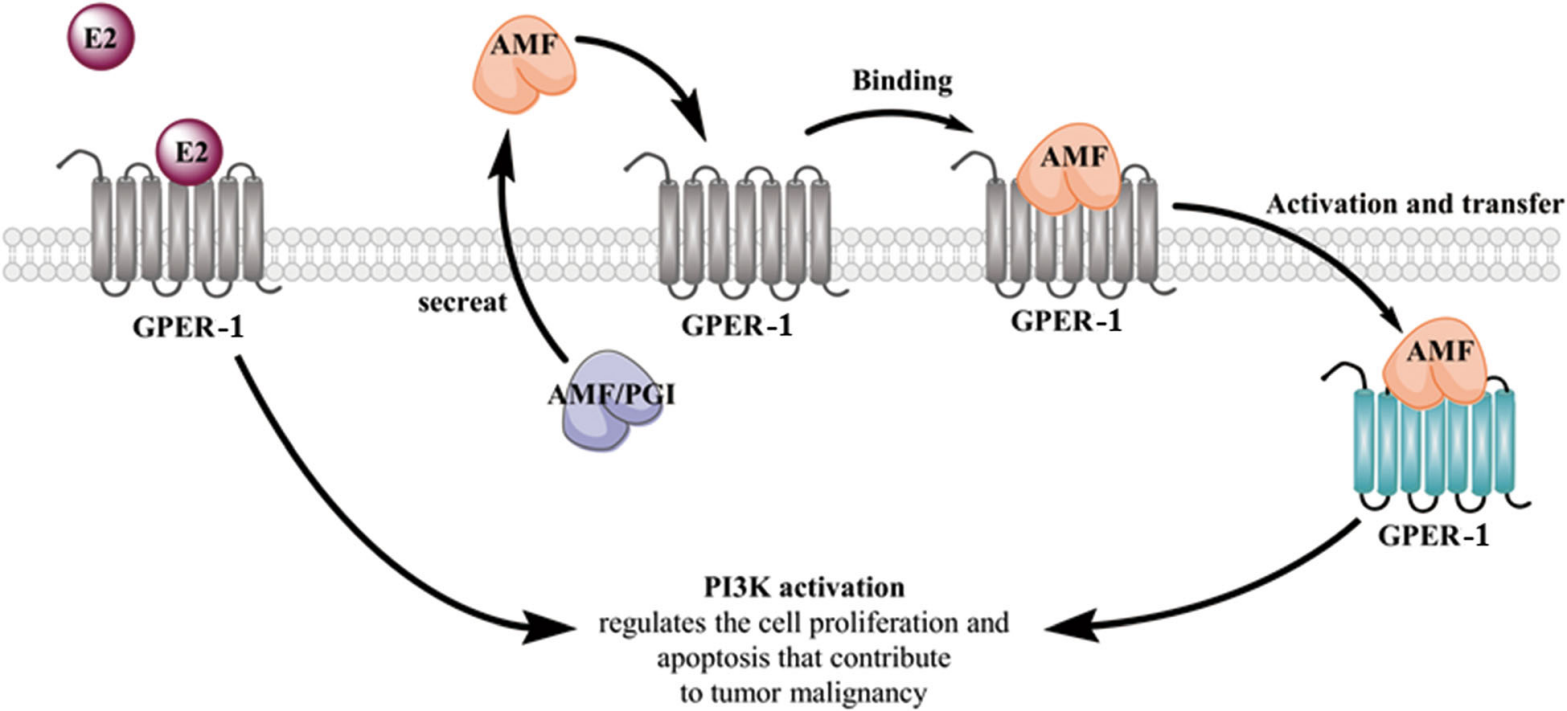
Schematic representation of AMF secretion and interaction with GPER-1, which drive EC progression. AMF is secreted through a non-classical mechanism and interacts with GPER-1, which results in GPER-1 activation and translocation. AMF binding with GPER-1 activates the PI3K signaling pathway, which enhances cell growth and suppresses cell apoptosis. The interaction between AMF and GPER-1 may contribute to tumor malignancy and provide targeted therapy for the treatment of EC

Our study uncovers a novel mechanism that AMF directly binds to GPER-1, and subsequently activates PI3K signaling pathway, which in turn accelerates the endometrial cancer cells growth. Furthermore, a positive clinical correlation between AMF and GPER-1 was found in endometrial cancer (Fig. 6). Thus, we provide the first piece of evidence that GPER-1 interacts with AMF and the complex contributes to endometrial cancer progression in animal and human histological experiments. So we propose AMF might serve as a novel therapeutic target in patients with GPER-1 positive EC.

## Additional file

Additional file 1: **Figure S1.** Y2H assay to detect the AMF-GPER-1 interaction. **Table S1.** Target guide sequences for GPER-1 and AMFR. **Table S2.** Oligonucleotides used for the RT-qPCR analyses. **Table S3.** Residue information of AMF and GPER-1 interaction. **Figure S2.** Protein and mRNA expression analyses of shGPER-1. **Figure S3.** Protein and mRNA expression analyses of cells overexpressing GPER-1. (DOCX 412 kb)

## Abbreviations

AMF: Autocrine motility factor
EC: Endometrial Cancer
GPER-1: G protein-coupled estrogen receptor 1
