# Supplementary material for: Autocrine motility factor promotes endometrial cancer progression by targeting GPER-1
Source: Cell Commun Signal. 2019 Mar 5;17:22. doi: 10.1186/s12964-019-0336-4 (PMC6402158; doi:10.1186/s12964-019-0336-4)
Supplement: Supplementary file 1 — Figure S1. Y2H assay to detect the AMF-GPER-1 interaction. Table S1. Target guide sequences for GPER-1 and AMFR. Table S2. Oligonucleotides used for the RT-qPCR analyses. Table S3. Residue information of AMF and GPER-1 interaction. Figure S2. Protein and mRNA expression analyses of shGPER-1. Figure S3. Protein and mRNA expression analyses of cells overexpressing GPER-1. (DOCX 412 kb) [file 12964_2019_336_MOESM1_ESM.docx]

**Additional file 1**

**Figure S1**

**
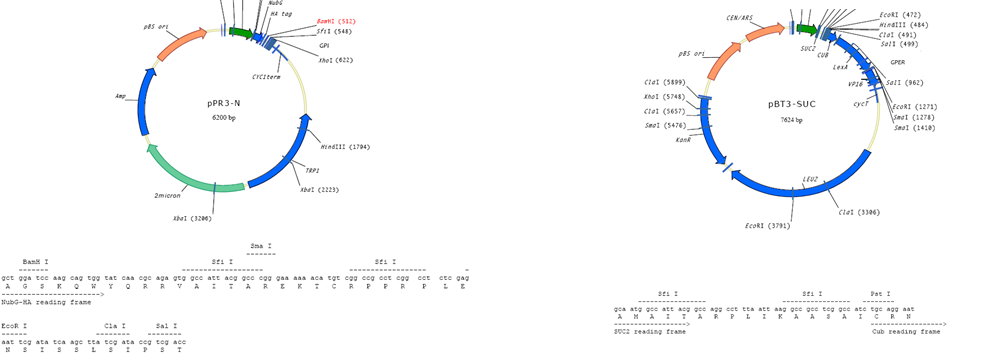
**

**Figure S1.** Y2H assay to detect the AMF-GPER-1 interaction. Bait plasmid PBT3-SUC-GPER-1-14 and prey plasmid PPR3-N-GPI-3 were cotransformed into the yeast strain NMY51, and a self-activation test was performed with selective plates.

**Table S1.** Target guide sequences for GPER-1 and AMFR

| **Gene editing** | **sequence** |
| --- | --- |
| GPER-1 (overexpression) | hGPER-1 cF: CACAGAATTCATGGATGTGACTTCCCAAGCCCGG  hGPER-1 cR: CACAGTCGACTCACACGGCACTGCTGAACCTCAC |
| GPER-1 (shRNA-1) | shGPER-1 F1:CCGGATGAGCTTCGACCGCTACATCCTCGAGGATGTAGCGGTCGAAGCTCATTTTTTG  shGPER-1 R1:AATTCTTTTTATGAGCTTCGACCGCTACATCCTCGAGGATGTAGCGGTCGAAGCTCAT |
| GPER-1 (shRNA-2) | shGPER-1 F2:CCGGATCGGCTTTGTGGGCAACATCCTCGAGGATGTTGCCCACAAAGCCGATTTTTTG  shGPER-1 R2:AATTCTTTTTATCGGCTTTGTGGGCAACATCCTCGAGGATGTTGCCCACAAAGCCGAT |
| AMFR (shRNA-1) | shAMFR F1:CCGGAGGAGAGAACTTGGATGAGAACTCGAGTTCTCATCCAAGTTCTCTCCTTTTTT  shAMFR R1:AATTCTTTTT AGGAGAGAACTTGGATGAGAACTCGAG TTCTCATCCAAGTTCTCTCCT |
| AMFR (shRNA-2) | shAMFR F2:CCGG CATGAGGTGCAACGTCGAATT CTCGAG AATTCGACGTTGCACCTCATGTTTTT  shAMFR R2:AATTCTTTTTCATGAGGTGCAACGTCGAATTCTCGAGAATTCGACGTTGCACCTCATG |

**Table S2.** Oligonucleotides used for the RT-qPCR analyses.

| **mRNA** | **Primer sequence** |
| --- | --- |
| GPER-1 | Sense: 5′-GCCTGCCGCTGCAGGAAACATTTC-3′  Antisense: 5′-CTCGGCGGTGCGCTAGCTAGCTCAG-3′ |
| AMFR | Sense: 5′-TTCGTCGGCACAAGAACTATC-3′  Antisense: 5′-GCACAGTCGTCATTGTTGACAG-3′ |
| β-Actin | Sense: 5′CAGCCATGTACGTTGCTATCCAGG-3′  Antisense: 5′AGGTCCAGACGCAGGATGGCATG-3′ |

**Table S3.** Residue information of AMF and GPER-1 interaction

| Residue  (GPI) | Atom  (GPI) | Residue  (GPER-1) | Atom  (GPER-1) | Type | Distance  (Å) |
| --- | --- | --- | --- | --- | --- |
| THR190 | N6569 | SER325 | O1519 | H-bond | 2.69 |
| THR190 | O6574 | SER325 | N1514 | H-bond | 2.87 |
| GLN198 | O6889 | THR364 | O1580 | H-bond | 2.54 |
| LYS194 | O4670 | GLU89 | N1552 | H-bond | 2.24 |
| THR542 | O4496 | PHE68 | O4319 | H-bond | 2.74 |
| ALA193 | N6806 | HIS353 | C1543 | ionic-bond | 1.45 |
| ALA197 | O6851 | VAL359 | O1571 | ionic-bond | 1.27 |
| GLN198 | C6862 | ILE360 | O1576 | ionic-bond | 0.88 |
| GLN198 | C6862 | ILE360 | C1575 | ionic-bond | 1.49 |
| LEU199 | C6853 | VAL359 | O1585 | ionic-bond | 1.26 |
| TRP228 | C6823 | ALA355 | N1813 | ionic-bond | 0.87 |
| GLN374 | C4889 | PHE117 | N2997 | ionic-bond | 1.44 |
| HIS389 | C5439 | TRP182 | O3100 | ionic-bond | 1.5 |
| HIS389 | C5440 | TRP182 | O3100 | ionic-bond | 1.4 |
| SER541 | C6469 | LEU311 | O4313 | ionic-bond | 1.67 |
| SER541 | C6469 | LEU311 | C4312 | ionic-bond | 1.63 |
| LEU545 | O6497 | SER315 | C4340 | ionic-bond | 1.22 |

**Figure S2**


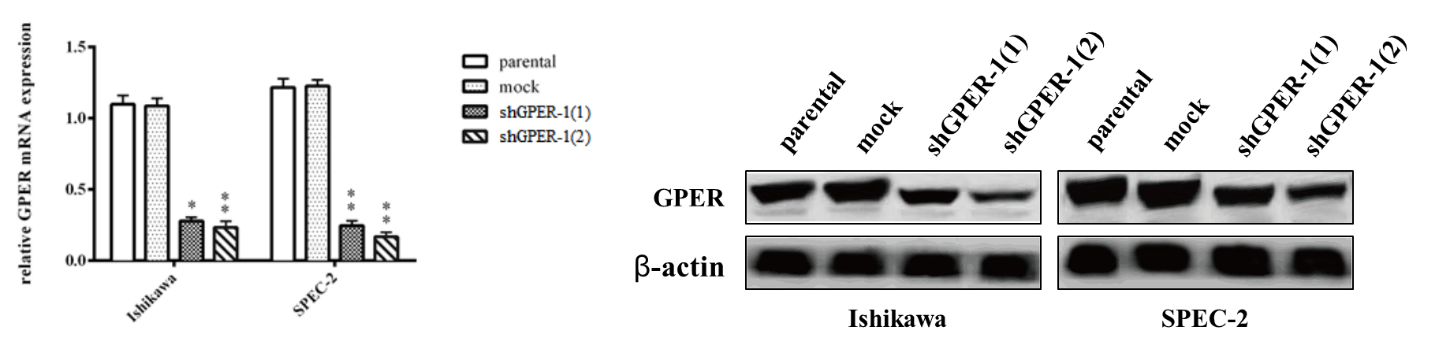


**Figure S2.** Protein and mRNA expression analyses of shGPER-1. Ishikawa and SPEC-2 cells were stably transfected with plasmid containing GPER-1-specific shRNA (shGPER-1(1) or shGPER-1(2)) or control plasmid (mock). Cells were then analyzed by qRT-PCR, and immunoblot analysis was performed to evaluate GPER-1 and β-actin protein levels.

**Figure S3**


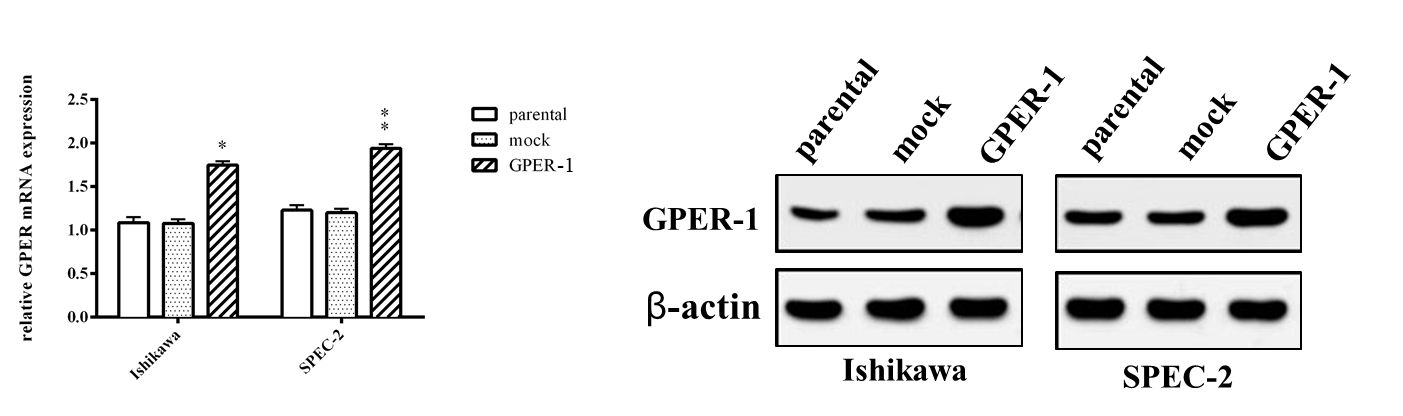


**Figure S3.** Protein and mRNA expression analyses of cells overexpressing GPER-1. Ishikawa and SPEC-2 cells were stably transfected with plasmid containing GPER-1-hRNA (hGPER-1) or control plasmid (mock). Cells were then analyzed by qRT-PCR, and immunoblot analysis was performed to evaluate GPER-1 and β-actin protein levels.
